# Searching synergy between esport and academy: the role of sport faculties and departments in CEE countries

**DOI:** 10.3389/fspor.2025.1539483

**Published:** 2025-04-15

**Authors:** Petra Hospodková, Jan Šíma, Vladimír Rogalewicz, Zdeněk Ledvina, Jakub Čubík, Daniel Opelík

**Affiliations:** ^1^ Department of Biomedical Technology, Faculty of Biomedical Engineering, Czech Technical University in Prague, Kladno, Czechia; ^2^ Department of Sport Management, Faculty of Physical Education and Sport, Charles University, Prague, Czechia; ^3^ Academic Department of Esport, Faculty of Physical Education and Sport, Charles University, Prague, Czechia; ^4^ Department of Telecommunications, Faculty of Electrical Engineering and Computer Science, VSB – Technical University of Ostrava, Ostrava, Czechia

**Keywords:** esports, stakeholder management, esports development, esports education, university, Central and Eastern Europe

## Abstract

**Objective:**

To identify the current challenges and barriers within the esports environment in the Central and East European countries and to explore the potential for collaboration between universities, particularly sports faculties, and the esports ecosystem. The research also aims to clarify the perceived roles of universities, including their research domains, within the context of esports.

**Methods:**

The research is based on a qualitative approach. Semi-structured interviews were used in the qualitative research to analyze attitudes toward the esports issues. The interviews were conducted with 18 stakeholders, the recordings were transcribed verbatim, coded and analyzed using the MAXQDA Analytics Pro software.

**Results:**

The analysis led to the following findings: Universities can collaborate with esports teams, players, and other stakeholders to research and analyze players' physical state and performance, offering courses and certifications for coaches and managers of esports teams, where they would learn gaming skills and player's health and physical preparation. Moreover, universities can facilitate interaction between esports teams and experts in the field of nutrition, sleep, etc., potentially leading to improved health and performance of players. Finally, universities can collaborate with esports associations to establish standards and licensing requirements for coaches, managers, and other professionals in the esports industry.

**Conclusion:**

The paper advocates collaboration between universities and esports organizations and encourages flexibility and innovation. Recommendations include establishing formalized training programs for players, coaches, managers and organizers, promoting esports within the university environment, fostering positive perceptions, and supporting university leagues.

## 1 Introduction

Esports is a form of a competitive practice using video games. It is often held as an organized multi-player competition with professional players playing individually or in teams. The growth of esports has appeared with the expansion of broadband Internet networks after 2000, and esports has turned into an entire industry including players, teams, leagues and tournaments, viewers and fans, media coverage, a special TV channel, the betting sector, special hardware producers. In recent years, esports has become an important part of the entertainment and gaming industry, especially among the younger generation.

Esports is totally considered a mainstream sport in 2024 (1). Estimated revenue in the esports market reached US$3.96bn in 2023 and is expected to show an annual growth rate of 8.21%, resulting in a projected market volume of US$5.43bn by 2027 (2). In 2024, there are expected to be over 285 million frequent viewers of esports worldwide, as well as some 291.6 million occasional viewers, when Asia-Pacific region has over 57% of the total esports viewers and Europe has around 16% of the global share of esports viewers; 76% of esports fans devote more time to esports than traditional sports (1, 3). Johan Sundstein, better known as N0tail, has been the highest earning male esports player. He has so far earned around $7.18 million from his esports ventures (February 2024) (3).

Out of the estimated global revenue of the esports market nearly US$ 4 billion, the figures for Central and East European (CEE) countries are very modest (in million US dollars): Austria 24.7, Czechia 24.1, Poland 14.4, Romania 13.09, Hungary 12.01, Lithuania 7.33, Serbia 5.29, etc. (4). On the other hand, the shares of people who watch esports are relatively high in these countries, e.g., Poland 29%, Austria 13%, Czechia 11% (5).

The current perception in the field of esports is quite diverse and affects the entire esports ecosystem, as well as the involved stakeholders. Recent research (6) has delved into the motivations behind viewership of live game streaming, emphasizing the importance of streamer interaction, platform functionality, and the comprehensive entertainment experience provided by OTT apps. This research suggests that modern media consumption behaviors, particularly through OTT platforms like Twitch, are reshaping viewer engagement and transforming the traditional viewer experience (6). As these platforms evolve, they influence not only how esports are consumed but also how stakeholders within the esports industry interact and depend on each other. The evolving media landscape requires that game developers, publishers, and tournament organizers adapt their strategies to capitalize on these new forms of viewer involvement.

Scholz (7) provide a comprehensive overview of the different stakeholders in the esports industry and their interrelationships. The author identifies several key stakeholders, including game developers, publishers, tournament organizers, teams, players, fans, media platforms, associations, agencies and betting platforms and describes the interrelationships between stakeholders as highly interconnected and interdependent. Vera and Terrón (8) describe the esports ecosystem as a three-level model, encompassing activities and actors ranging from those independent of esports to those that have emerged as a result of it. Users, such as professional players, coaches, and analysts, constitute the core of the system. Streamers and casters play a pivotal role in engaging with users, with their impact significantly mediated by the technological capabilities of the platforms they use, which supports dynamic interaction between the streamer and the audience (6).

Esports as a relatively new phenomenon raise many controversies, the central one being whether esports can be classified as a sport and eventually, for example, be included in the program of the Olympic Games (9–12). The general public has a mixed attitude towards esports and computer gaming as a whole, highlighting the health and psychological risks. Most esports generally require participants to sit and/or move little while playing, which raises concerns about a sedentary lifestyle by players (13). Other concerns relate to mental health and the potential for addiction (14). Nevertheless, the COVID-19 period with its lockdowns, curfews and expansion of online technologies led to a widespread convergence of sports and esports (15). In September 2021, the Olympic Council of Asia announced eight esports games would officially debut as medal sports for the 2022 Asian Games in Hangzhou, China (16). In July 2024, the International Olympic Committee (IOC) decided to create Olympic Esports Games. The first edition will be held in 2025 in the Kingdom of Saudi Arabia (17).

All of the above also applies to CEE countries. This region has a quite high number of esports viewers, however it is not so successful from the economic point of view, when esports revenues are rather negligible in this region. On the other hand, it has proved to be quite progressive in converging esports and traditional sport ecosystems. The Polish Esports Association announced that it was accepted as a member of the Polish Olympic Committee in March 2023 (18). Similarly, E-sport Federation of Slovenia has become a member of the Olympic Committee of Slovenia—Association of Sports Federations in June 2022 (19). In general, the entire ecosystem of esports in the CEE countries requires much stronger institutional backing and support for its different parts. This must start with research into the views of all stakeholders.

The CEE countries have a long tradition of sports faculties or departments of traditional universities. There is a strong potential of know-how and human resources experienced in all kinds of support for traditional sport. These resources can form the basis of esports-focused research and engagement. The authors see a great potential in the synergy and cross-fertilization of these teams and the esports ecosystem, which would contribute to the development of both the esports field and traditional sport. Thus, sport faculties may play a decisive role in the esports industry development. To test this hypothesis, we conducted the research presented here in the Czech Republic as a representative of the CEE region. This geographical location was chosen due to the Czech Republic's unique position within the CEE region. The country not only has a well-developed esports community but also benefits from strong governmental support and a high level of international experience among its stakeholders. These factors make it an ideal representative for studying the potential and challenges of integrating traditional sports faculties with the esports ecosystem. According to Omnicom Media and the Czech News Center research (20), the number of Czechs watching and playing esports, gaming or playing mobile games is estimated at 3.4 million people, which is more than 60% of the population aged 15–50.

The primary objective of this study is to identify the current challenges and barriers within the esports environment in the CEE countries and to explore the potential for collaboration between universities, particularly sports faculties, and the esports ecosystem. This research also aims to clarify the perceived roles of universities, including their research domains, within the context of esports.

## 2 Methods

To precisely explore the role of stakeholders in the esports ecosystem, it is very important to approach all groups of stakeholders who have influence or power in the particular environment, and include their opinions. Therefore, a qualitative approach was chosen to explore this issue.

### 2.1 Stakeholder selection

In the first phase, a map of potential stakeholders was compiled, which was divided into nine basic sets. The authors used purposeful and snowballing sampling (21) to select stakeholders they approached to participate. The specific stakeholder representatives who came forward were sampled based on seniority and function with preference given to senior representatives with a history of direct involvement in esports. All stakeholder representatives were contacted via a standardized email invitation. A total of 20 stakeholders were contacted, of which 18 agreed to participate.

The interview script was prepared that focused on the following key areas:

• Perceived challenges and barriers in esports;
• Collaboration between esports and sport faculties/universities;
• The role of sport faculties/universities.

Semi-structured interviews were used in the qualitative research (see Appendix A) to analyze attitudes toward the esports issues.

**Figure 1:**
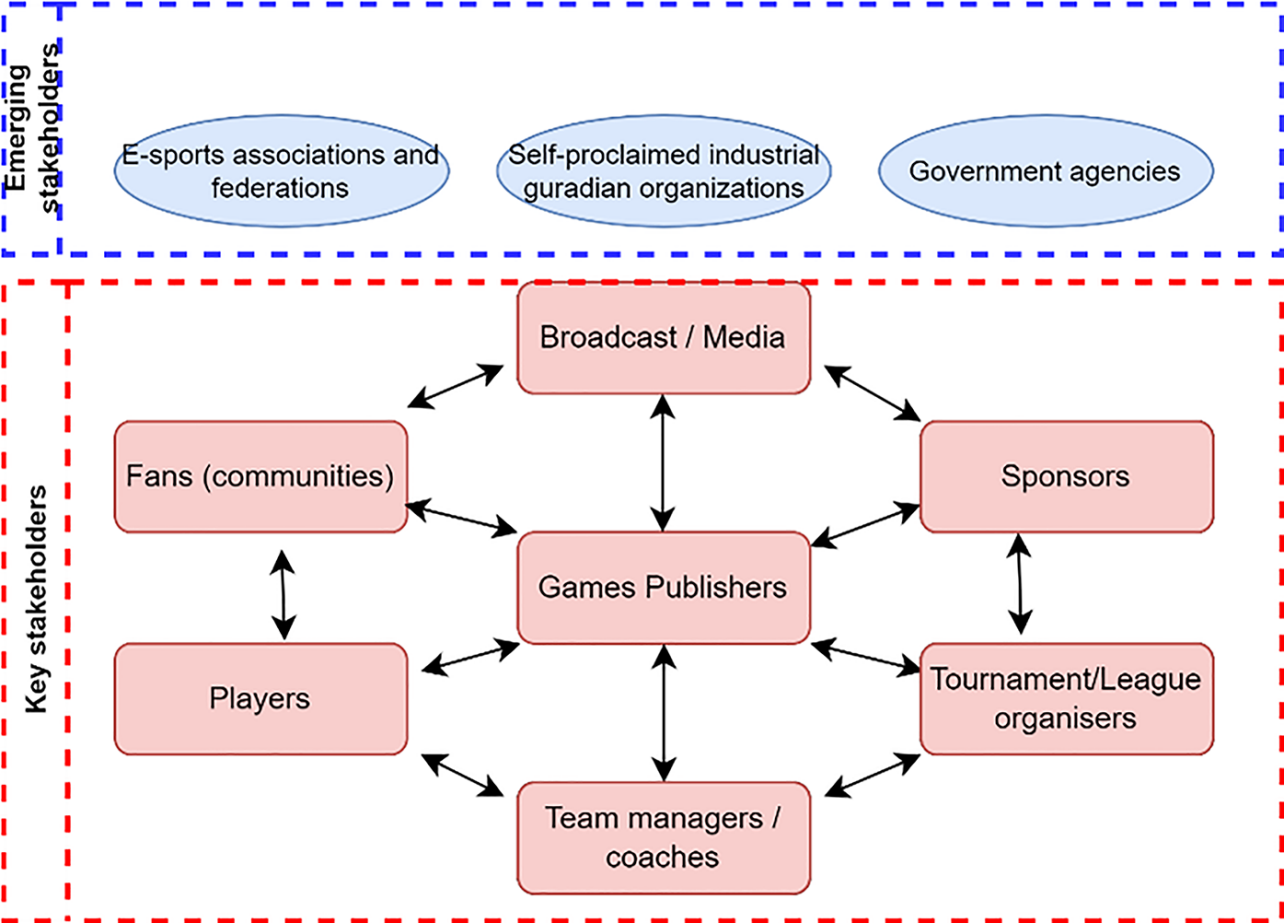
Overview of the key stakeholders in the esports network.

The selection of stakeholders was based on the model of Peng et al. (22), adapted for the Czech Republic (see Figure 1).

### 2.2 Recruitment and consent process

The participants were recruited using publicly available contact details, and the initial contact was made either via standardized email. The purpose of the study, its scope, and ethical considerations were clearly outlined in the preliminary communications. An informed consent was secured verbally at the commencement of each interview session, which was digitally recorded using MS Teams. This approach not only facilitated a reliable method of obtaining consent, but also ensured that all participants were fully aware of their involvement and the objectives of the study.

Before the actual interviews, each participant received a detailed information package by email. This package included an attachment that described the objectives of the study, the nature of the questions to be asked, and the ethical safeguards in place, including the anonymization of data and the right of participants to withdraw from the study at any time without penalty.

### 2.3 Interview methodology

The interviews were conducted by a team of three experienced interviewers, each using MS Teams to ensure consistent audio quality and enable secure recording. The average duration of the interviews was approximately 35 min, optimizing the depth of dialogue while respecting the participants' time. This duration was sufficient to explore the thematic areas of interest deeply without causing fatigue or disengagement among the participants. After each interview was completed, the recordings were transcribed verbatim. Strict measures were taken to anonymize all transcripts, systematically removing any information that could potentially identify the participants. This included specific names, titles, or any other unique identifiers, thereby upholding the stringent confidentiality requirements of the research.

### 2.4 Analytical framework and coding process

The analysis of the interview transcripts followed a structured content analysis methodology. The open coding technique was applied (23), initially independently by three analysts to ensure a comprehensive exploration of discrepancies and to enhance the validity of the interpretations. This phase of coding was iterative, continuing until data saturation was achieved—indicated by the emergence of no further new codes.

The first coding framework was developed through repeated listening and detailed reading of the interviews by the lead investigator. This initial framework captured the emergent themes and patterns in the data. Subsequently, a second version of the coding framework was collaboratively developed during sessions involving all analysts. This version was refined through joint reviews, where the analysts achieved consensus on the naming of codes, merged similar or overlapping codes, and removed any codes that were deemed imprecise or redundant. The final coding framework was rigorously tested for reliability and validity. Adjustments made during the review sessions included fine-tuning code definitions, ensuring that each code distinctly represented a specific aspect of the data. This iterative refinement process was critical to developing a robust analytical tool that accurately reflected the nuances of understanding and perspective captured during the interviews.

### 2.5 Data analysis using MAXQDA analytics Pro

The analysis of interview transcripts was facilitated by MAXQDA Analytics Pro software (version 24.0.0), employing its robust functionalities to enhance data interpretation. Key features used included:

• **Coding:** Creation and application of a sophisticated system of codes to organize data thematically.
• **Memos:** Use of the note editor for summarizing and highlighting significant observations.
• **Code frequencies:** Examination of the frequency and distribution of codes to assess thematic prominence.
• **Code relation browser:** Exploration of relationships between codes to identify overlaps and/or co-occurrences.

## 3 Nomenclature

The results of the esports ecosystem analysis allow us to analyze them under three basic headings defined in the methodological section. Namely, the challenges and barriers in esports, the collaboration between esports and sports faculties/universities, and the role of sports faculties/universities.

### 3.1 Challenges and barriers in esports

**Figure 2:**
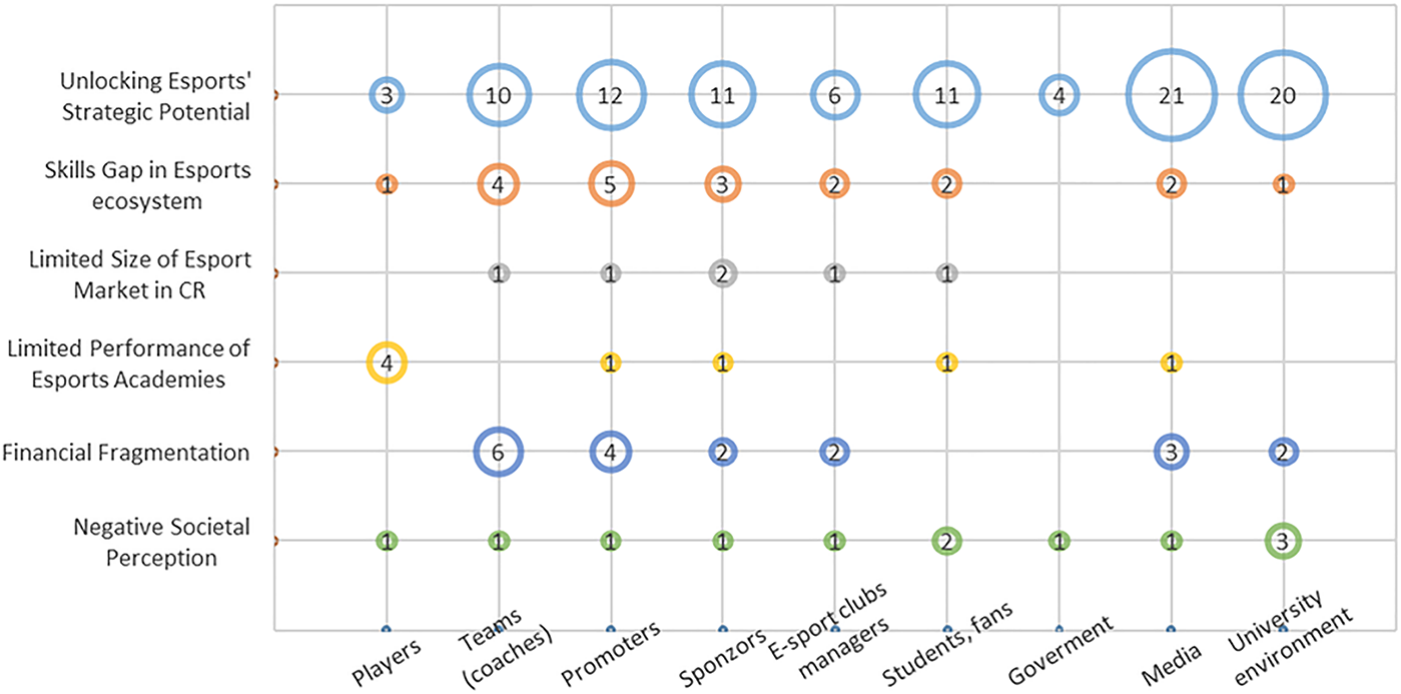
Challenges and Barriers in esport.

One of the research goals was to identify the general perceived barriers to the future development of the esports ecosystem (see Figure 2).

Stakeholders paid most attention to the strategic management issue coded as “*Unlocking esports strategic potential*” (specific qualitative analysis codes are printed in italics and shown in quotation marks). In summary, numerous groups and organizations are involved in esports management, making it challenging to unify them under one umbrella, similar to traditional sports. The development of esports in the country is still in the process, and there is a potential to integrate it into existing educational and sport structures, with schools being a suitable starting point due to the association with computers. However, effective management should involve tournament organizers, administrators, and other stakeholders, possibly through a commission or forum to establish common guidelines and principles. The institutional support for esports is currently mostly lacking, and a unified approach is required to navigate the challenges ahead. Within this code, it was highlighted that the fragmentation within the industry is a significant problem, and the need to bring different actors together to develop training materials to help each other, which is challenging given the lack of communication. As a result, there is currently an uncoordinated emergence of various organizations and associations, poor communication and competing interests that often hinder progress. While there are associations like the Czech Esports Association (CEA), not all teams are part of them, and concerns remain about their effectiveness and representation. Stakeholders also expressed the need for a centralised infrastructure with government support, particularly for project funding and establishing the foundations for the development of the gaming industry. In Czechia, they see the Ministry of Education, Youth and Sports and the Ministry of Health as the main possible initiators of this change.

One of the significant barriers to esports development is the “*Skill gap in esports ecosystem*”. The lack of formal education and specialized educational programs for players, coaches, managers, and organizers. Specifically, stakeholders noted deficiencies in leadership training for coaches, business management skills for team managers, and financial literacy among esports organizers. In particular, coaches often lack basic education in team leadership and psychological influence on players. Establishing specialized educational platforms for esports could overcome this barrier and contribute to an overall improvement in the esports ecosystem.

While esports has global potential, the CEE market is limited by its size–*“Limited size of esports market in the country*”. Collaboration with international institutions and exchange programs can help overcome this barrier and support the development of esports in the CEE countries. Market consolidation and the maintenance of strong entities with good strategies and finances are also essential for sustainable industry growth in the future.

“*The limited performance of esports academies*” has been identified as another discussed barrier. The ideal scenario would be for young players to have the opportunity to join academies, either as part of a university environment or under the umbrella of an association, and thus get the chance to collaborate with professional teams. This collaboration could be mutually beneficial–professional teams would have access to young talented players whom they could mentor and develop, while young players would gain valuable experience and a chance to make a mark. However, this ideal scenario has not been fully realized yet, and there are several challenges, such as how to ensure that young players are not financially disadvantaged while still receiving the necessary developmental opportunities. This barrier to the utilization of esports academies primarily concerns the amateur scene and the potential development of young talented players in the CEE countries.

Another barrier that emerged from the interviews was identified as “*Financial fragmentation: challenges faced by esports organizers*” with a particular emphasis on the teams (coaches) side. Financial resources represent another significant challenge as they are essential for genuine development but remain insufficient due to an excessive number of entities and the dispersion of sponsorship funds. The financing of esports within universities and higher education institutions also faces resource scarcity, as the costs often exceed the initial expectations of these institutions. The functioning and success of each stakeholder is often directly proportional to the number of partners and associated funding. Currently, there is no effective model for monetizing viewers, neither at the player level nor within organizations.

The last code in this category that was consistently mentioned by all stakeholders was “*Negative societal perceptions of esports and its acceptance as a sport*”. In Europe, esports still faces negative perceptions and scepticism, probably due to a lack of understanding of how esports works. Another issue is the absence of official recognition of esports as a sport by the governments, creating an uncertain environment for esports organizations. It will take time and effort to gain broader societal acceptance of esports and to integrate it into university sports management systems (Figure 2).

### 3.2 Collaboration between esports and sport faculties/universities

**Figure 3:**
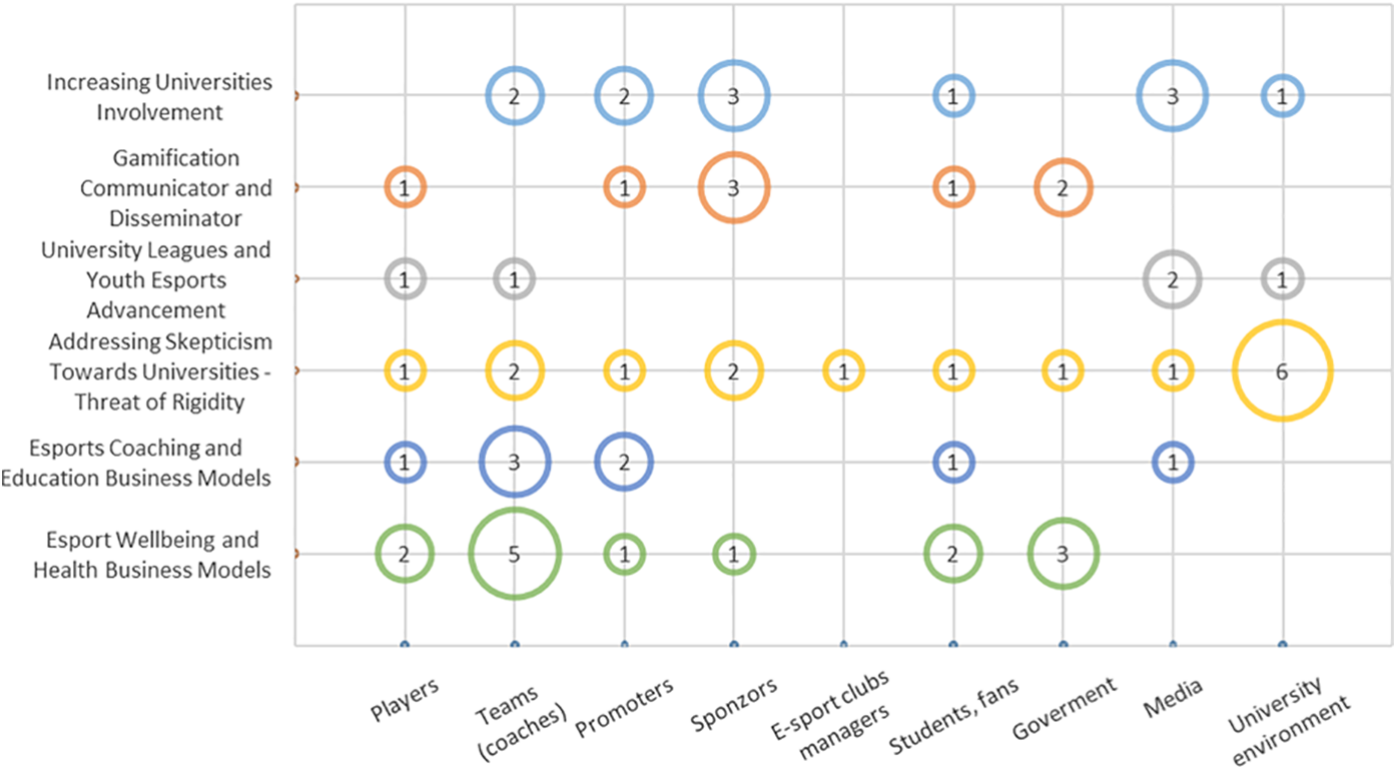
Possibilities of collaboration between sports-oriented faculties and the esports environment.

In this area, the study explored the factors that hinder or promote collaboration between sports-oriented faculties or university departments and the esports environment (see Figure 3).

A positive finding is that most stakeholders perceive the “*Increasing involvement of universities*” in the esports ecosystem. In the past two years, there have been significant changes in the involvement of universities in the esports environment in the country. Previously, universities were seen as insignificant players in esports, but now they are actively engaged through university leagues and partnerships with leading entities. One informant directly stated: “*People perceive the academic world as a guarantee of quality and seriousness*,” while another said: “*For organizers, the greatest added value could be media coverage and research. Suppose there were data on how esports can benefit players. In that case, we can communicate the positive impacts of esports, such as improved reflexes, memory, or English language skills, and that's why the involvement of universities is welcome*”.

Sponsors and government representatives, particularly, emphasized the potential role of sports faculties as “*Gamification communicators and disseminators*”, aiming to enhance communication and information dissemination among esports stakeholders. This role involves distributing educational materials about esports for parents and children at offline events. Gamification and modern technologies are increasingly crucial in the esports ecosystem. Sports faculties can act as vital intermediaries among various esports stakeholders, supporting the growth. One informant stated: “*We should explore the possibility of researching to examine the impact of gaming on personal development. It's important to focus on whether gaming can effectively help prevent future issues, such as substance abuse, or similar concerns. Gaming allows us to engage with young individuals and make a meaningful impact directly, especially when we combine it with appropriate educational efforts. There's no doubt that gaming can foster a range of competencies that are relevant to their future professional lives.*”

The additional, repeatedly mentioned issue is represented by the “*University leagues and youth esports advancement*” code. Czech stakeholders appreciate the current organization of academic leagues and tournaments and emphasize the need for further support in creating events that would attract the attention of young players. To achieve higher university involvement in academic leagues, it is essential to establish formalized training programs for players, coaches, managers, and organizers. Promoting esports within the university environment and fostering positive perceptions can also help. They emphasize the importance of creating stories and narratives around esports teams and players to increase interest and engagement.

A recurring theme across interviews, particularly among university stakeholders, was “*Addressing skepticism towards universities–threat of rigidity*”. Even though there is interest from commercial partners in collaborating with universities, it is often influenced by the inflexibility of processes at the university/faculty level. This inflexibility manifests itself in both organizational and administrative aspects. Younger and more dynamic companies often have greater flexibility than traditional ones and choose to collaborate with private organizations. One stakeholder also stated: “*So far, we've had the impression that there is little to no interest in esports in the university environment. The main problem has been their unwillingness or lack of interest.*” Another problem lies in the mutual communication and establishment of rules between universities and esports organizations. While universities have their internal regulations, esports organizations have their own rules often developed in connection with their partners and sponsors. Complications arise when these rules and contracts clash, which can lead to a cancellation of potential events or collaborations. One stakeholder provided an example: “*Esports organization partners have a contractual obligation that their brand has to be part of any event or broadcast. This may conflict with university regulations because these partners are not official sponsors.*”

The last two areas focus on the possibility of creating exciting business models between universities and the esports environment. There are two codes “*Esports coaching and education business models*” and “*Esports wellbeing and health business models*” that have emerged from the synthesis of information from the interviews. Interestingly, university stakeholders do not express their views on this topic. The following areas can serve as a basis for the conceptualization of business models.

• **Physical preparation:** Universities can collaborate with esports teams, players, and other stakeholders to research and analyze players' physical state and performance.
• **Education for coaches and managers:** Universities can offer courses and certifications for coaches and managers of esports teams, where they would learn gaming skills and player's health and physical preparation.
• **Physiotherapy and health care:** Universities can provide physiotherapy services and expert advice on the ergonomics of gaming equipment and maintaining healthy lifestyle for players. Universities can facilitate interaction between esports teams and experts in the field of nutrition, sleep, etc., potentially leading to improved health and performance of players.
• **Establishment of standards and licensing:** Universities can collaborate with esports associations to establish standards and licensing requirements for coaches, managers, and other professionals in the esports industry (Figure 3).

### 3.3 The role of sport faculties/universities

**Figure 4:**
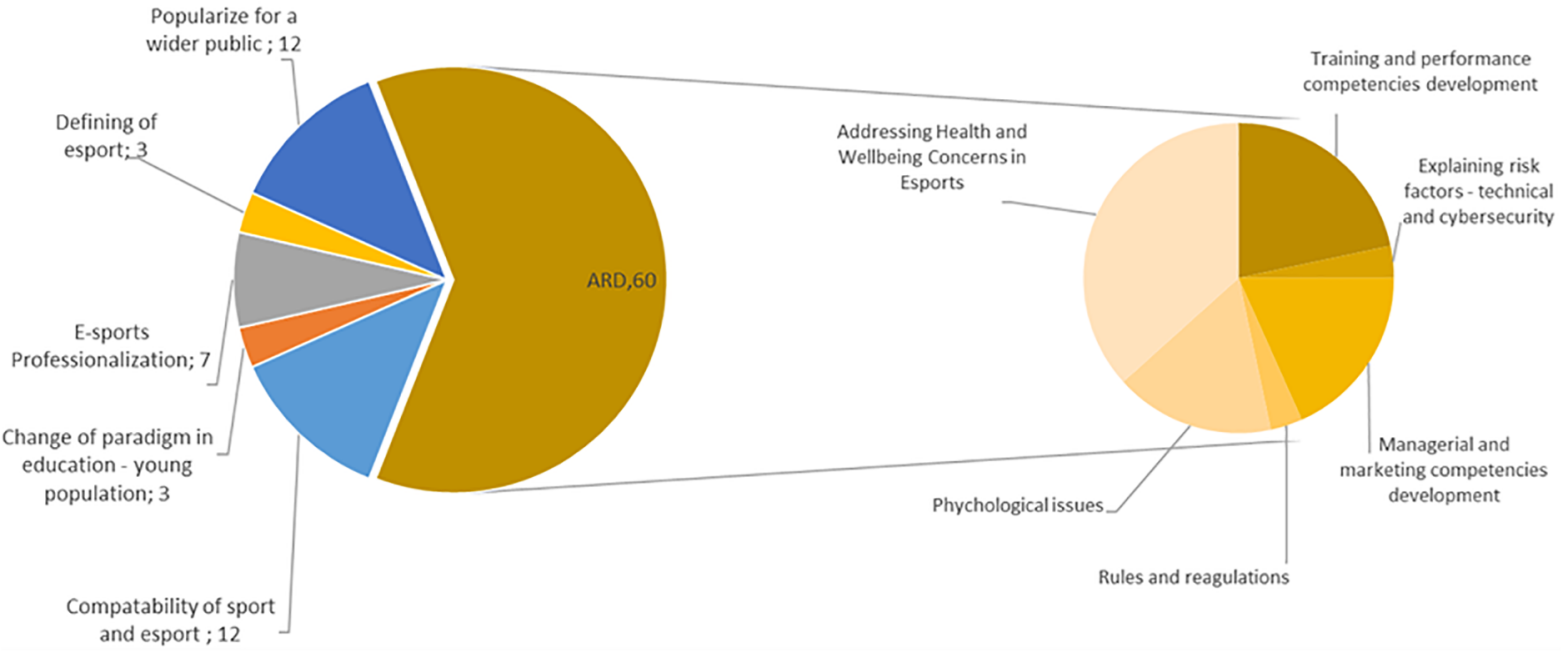
Perceived roles of universities, including research areas.

Universities, sport faculties and/or university departments can support various stakeholders or even take on their role. Applied research plays a pivotal role in multiple esports domains. However, what is noteworthy is the perceived need to foster synergy between traditional sports and esports. Another aspect is the definition of esports itself. Despite the esports industry experiencing rapid growth and being considered highly popular, the universities should act as an intermediary between the general public and the esports ecosystem to promote a positive perception of this field (Figure 4).

## 4 Discussion

Esports can be characterized as the “top level of video gaming in terms of skill and professionalization” (24). The field of professional video gaming has been expanded in terms of interaction between the computer and human. Owing to areas focusing on professional-level computer work, such as esports, the time spent consuming video game content can amount to up to half of the day, seven days a week (25, 26). This content and its extent can influence a person positively as well as negatively. In recent years, esports have become an important part of the entertainment and gaming industry, especially among the younger generation. Studies indicate multiple perspectives from which esports can be viewed, including competitive play, structured esports events, professional conduct, and its acceptance as a bona fide sport (6, 7, 9, 13). This development is further complemented by a significant role played by live-streaming platforms, which not only serve as a primary medium for viewing but also enhance the interaction between fans and the sport. Meng-Lewis et al. (27) claim that live-streaming platforms like those used for the King Pro League improve the fan experience by meeting and often exceeding their expectations regarding ease of use and engagement, which subsequently influences their continuous usage intentions. This unique trend is too significant to overlook or undervalue; nevertheless, it is imperative to examine its capabilities and applications beyond its primary concentration. The development of the esports landscape differs from country to country, influenced by the level of institutional backing and strategic oversight (28, 29).

The contemporary landscape of the esports ecosystem was delineated by Werder (30) affirming its dynamic nature and continual evolution. A number of studies have addressed the question of whether the esports community or its ecosystem needs more institutional strengthening (31, 32). They examine the possibility of increasing the formal organization internally and assess if the current esports infrastructure is sufficient. There is also a look at the potential benefits of creating new business models in this field. It is essential to point out that to tackle these intricate issues, one must grasp the attitudes and opinions of essential stakeholders in the esports ecosystem.

The esports industry in Central and Eastern Europe (CEE) faces diverse challenges and opportunities in organization and management. This emerging ecosystem requires robust frameworks to address issues of regulation, stakeholder collaboration, and sustainable growth. Poland and Slovenia have led the way in institutionalizing esports by integrating their national esports associations into Olympic committees (18, 19). This institutional support has fostered greater stakeholder collaboration between government bodies, teams, and event organizers. Scholz (7) highlights the importance of strong interconnections between key stakeholders such as game publishers, tournament organizers, and media platforms to create a resilient esports ecosystem. CEE countries face fragmented financial ecosystems, with limited regulatory oversight. For instance, studies in Hungary and Romania reveal exploitative contracts that hinder player development and professional management (33). Moreover, the rapid rise of esports betting introduces ethical concerns that require stringent governance, especially to protect younger audiences (34). Hungary and Poland are pioneering educational programs aimed at bridging gaps in managerial skills, including team leadership and strategic oversight (35–37). Universities are playing a critical role by creating esports academies and research initiatives that train managers, coaches, and players in professional competencies (30, 38). Poland's Intel Extreme Masters in Katowice exemplifies how events can integrate advanced fan engagement strategies, such as real-time analytics and virtual reality zones, to enhance audience experiences and stakeholder alignment (39). These innovations not only boost economic sustainability but also strengthen organizational cohesion.

While Poland (40) and Hungary exemplify sophisticated management models, countries like Bulgaria and Estonia, Serbia and Slovenia (41) are beginning to explore collaborative frameworks. Such initiatives could benefit from shared resources and expertise to standardize operations and expand market reach (32). Scholz (7) underscores the potential of regional alliances in mitigating market fragmentation and fostering unified growth.

Our survey sought the views of esports stakeholders and was conducted in Czechia as a typical representative of the situation in the CEE countries. The results of the research above all revealed deficiencies in strategic management in esports. National umbrella organizations face a number of challenges, in particular the lack of institutional support, the presence of multiple bodies involved, the lack of communication between them and the overall fragmentation of the sector. These issues impede the ability of the associations to effectively manage the industry. The current scenario, where some clubs see no necessity to be members of an umbrella association, diminishes its authority and strategic management capabilities.

Regulation and a unified direction in the esports sector are being sought by the European Union (42–44). Due to the general influence of the video game industry on human-computer interaction, and particularly its impact on children and adolescents, the creation of a stable ecosystem with clearly defined rules is necessitated (45, 46). In the creation of this ecosystem, the academic sector, and especially public state universities, should play a key role.

In professional sports, it is a common practice for national associations to take charge of organizing and regulating sporting activities (47). These associations and federations play a pivotal role in formulating rules and standards for the sport. Clubs, by registering with these entities, commit to adhering to these established standards, ensuring uniformity and fair play. Following a similar approach, esports associations should organize the most significant esports competitions with only registered clubs eligible to participate in these events. Furthermore, the association should serve as the sole point of communication with international authorities such as the International Esports Federation, Global Esports Federation, or World Esports Association. Peng et al. (22) gives some examples of such national association outside the CEE countries, however, generally he is rather critical. Establishing a centralized and standardized system would enhance the associations' effectiveness in managing the esports industry, promoting consistency, and strengthening its relationships with international entities.

Associations in cooperation with universities can provide educational programs for players, coaches or managers. The cooperation with universities can include the creation of academic modules, workshops, mentoring programs, certified coaching courses and internship opportunities in partnership with esports organizations. This would also address the skill gap often mentioned by informants. This is characterized by a lack of formalized education and professional programs for players, coaches, managers and organizers in esports in the CEE countries. The most commonly identified gaps are poor communication skills, team leadership skills and generally lack of professionalism across the sector. This problem is not unique. It also appears in other new sports, where the organization is often in the hands of enthusiasts who are more players than coaches or managers at the beginning. It is in new sports that the education of coaches and other professionals is crucial. In the case of traditional sports, it is common to follow the well-established model of a sport federation-university cooperation (48). The question remains how universities (most often their sports faculties) will handle the differences between traditional sports and the skills required in esports.

As proposed by Scott and his colleagues (38), courses tailored for esports players could integrate theoretical knowledge encompassing the history and development of the esports field, along with insights into its socio-cultural context. Additionally, these courses could provide practical skills, including strategies, tactics, and effective team communication. Emphasizing health and wellness aspects within these programs would educate players on the importance of looking after their physical and mental well-being. Integrating esports with the university environment would probably increase the prestige of the industry in society and address the negative societal perception of esports and its (non-)acceptance as a sport. Esports is often considered by the public as passive computer sessions. However, it is important to emphasize that these activities offer a great opportunity to develop skills and knowledge that are crucial for personal development (49). Moreover, it can be foreseen that the university environment, where young people with IT skills are concentrated, can play a key role in the development of esports in the future. According to the responses of the informants, esports associations seem to perceive the university environment also as a potentially attractive setting for the engagement of young players in their structures.

Darcy et al. (50) presented compelling evidence and emphasize the priority of creating strong bonds among club members within an organization. This aspect significantly reinforces the deep sense of belonging and mutual support among club members, from volunteers to board members. Strong ties create a solid foundation for developing the supportive capital associated with the local, regional and national stakeholder communities connected to the organization. This is also the reason why stakeholders identify significant potential in university leagues. They commend the ongoing efforts of some universities to organize university tournaments and emphasize the necessity for additional support for similar events. These events have the potential to capture the attention of young players and foster the creation of a shared community. Survey interviews showed interest in collaborating with universities, but concerns arose about the inflexibility of formal processes at the university or faculty level. Chidziwa et al. (51) pointed out that the excessive bureaucracy and administrative burdens in a rigid academic setting can impede innovation. Processes such as approving new curricula and introducing new technologies are often lengthy and complex, especially compared to smaller, more dynamic companies that are more flexible and adaptable.

**Figure 5:**
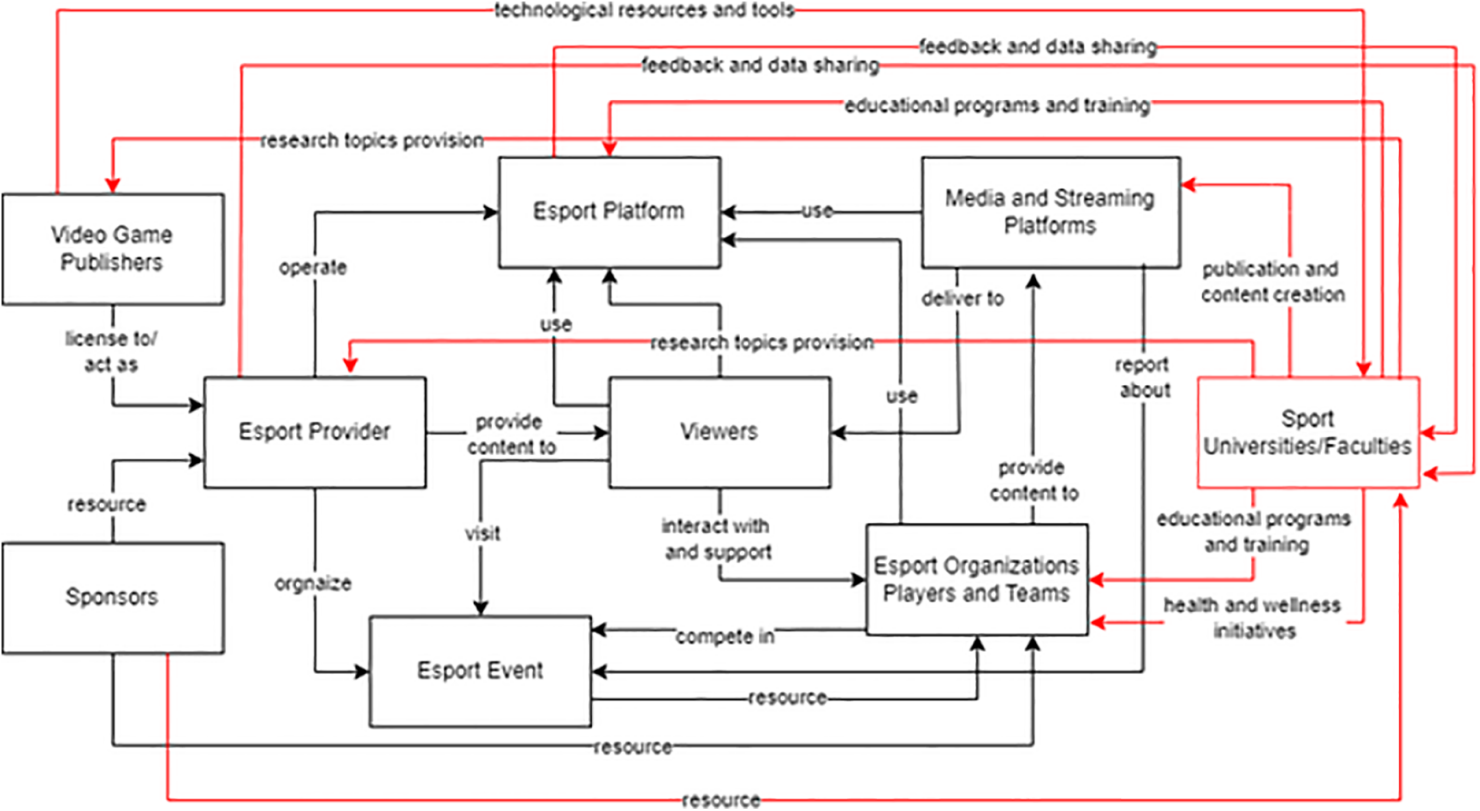
University assimilation model in the esports environment.

Addressing the inherent inflexibility within the university environment is crucial for the institution to proactively meet the evolving needs of the esports community, the demands of the labor market, and the expectations of all stakeholders in the field. Implementing effective strategies can empower universities to establish a dynamic and adaptable environment that appeals to players, fans, associations, tournament organizers, game developers, and other stakeholders. The results suggest and recommend that university environments engage in key activities related to the education of esports professionals, organization of esports competitions and research in the field of esports, highlighting the potential societal benefits of esports. The involvement of universities in the esports ecosystem thus offers a number of potential advantages and benefits not only for the esports actors, but also for the universities themselves. These observations create a link to the esports ecosystem as characterized by Werder (30) and point to its status as an emerging industry. The findings of this survey therefore facilitate the adaptation of the model for implementation in academic institutions specializing in sport education. The enriched model [see Figure 5, based on (30)] delineates the dynamic function of the university within the esports ecosystem, accentuating the mutual engagement and reciprocal influence therein. It elucidates the multifaceted role of universities that not only spread knowledge and cultivate talent through research and educational activities but also integrate industry insights and resources to support esports innovation and health initiatives (Figure 5).

Practical management implications of these findings are significant for both esports organizations and academic institutions. By establishing formalized training programs and fostering collaboration between universities and esports entities, stakeholders can ensure a more structured and professional approach to esports management. This includes developing specialized educational platforms for coaches, managers, and players, which can bridge the current skill gaps and enhance the overall professionalism within the industry. Additionally, universities can play a pivotal role in conducting research that informs best practices in player health, performance, and well-being, thereby contributing to the sustainable growth of the esports ecosystem.

### 4.1 Strengths and limitations of the study

This study stands out as a pioneering effort to explore the potential for collaboration between universities and the esports ecosystem in CEE. Its comprehensive qualitative approach, based on interviews with 18 diverse stakeholders, provides a rich, multi-faceted understanding of the opportunities and challenges within this rapidly evolving field. The focus on the Czech Republic as a representative of the CEE region ensures the findings are both contextually grounded and regionally applicable. Moreover, the study highlights actionable recommendations for fostering synergies between academia and esports stakeholders, addressing critical gaps in research, education, and institutional support.

Despite its strengths, the study also has certain limitations. While the sample reached theoretical saturation, some minority groups of stakeholders in relation to esports may have been inadvertently underrepresented. This could mean that perspectives unique to smaller or less visible actors were not fully captured. Furthermore, the semi-structured interview format, while allowing for in-depth and expansive discussions, sometimes resulted in responses that were broad in scope, potentially limiting the depth of analysis on specific aspects of university-esports collaboration.

Another limitation is the study's geographical focus on the Czech Republic. Although the country was selected as a model representative of the CEE region due to shared socio-economic and institutional characteristics, further research is needed to validate the findings across other CEE countries. However, this limitation is mitigated by the region's similar conditions, making the results broadly relevant. The Czech Republic's well-developed esports community and supportive governmental framework provide an ideal foundation for scaling insights across the CEE.

The choice to focus on the Czech Republic is supported by its role as a microcosm of the CEE esports ecosystem. The region's countries share comparable economic conditions, institutional structures, and historical contexts, which enhances the transferability of the findings. By identifying common challenges—such as market fragmentation, skill gaps, and the need for institutional backing—the study provides a robust framework for fostering esports development across the CEE. The potential for cross-border collaboration and resource sharing further reinforces the relevance of the proposed strategies to the entire region. This regional applicability underscores the significance of the study and positions it as a key reference for academics, policymakers, and industry leaders.

Finally, the broad thematic coverage of the research may have left some emerging trends or niche areas—such as technological innovations or the role of smaller esports organizations—underexplored. These aspects present valuable directions for future studies.

By addressing these limitations transparently while emphasizing the study's robust design and practical contributions, the article provides a compelling case for advancing research and practice in this field.

## 5 Conclusions

This article thoroughly examines the esports ecosystem in CEE countries and the Czech Republic as a model country, making it a valuable resource for academics, practitioners and policy makers. The CEE countries are considered to be a suitable closed ecosystem for the analysis of actors (not only) in the esports sector from an economic, technical, academic and policy perspective. In particular, the results on the coexistence and synergy between esports and universities are valuable and may well help to develop similar analyses for other countries around the world, especially for EU countries.

The article also highlights significant challenges, including strategic management, skills development, social perception and local market constraints, which are essential to formulating effective strategies and ensuring sustainable esports growth. Recommendations include establishing formalized training programs for players, coaches, managers and organizers, promoting esports within the university environment, fostering positive perceptions, and supporting university leagues. The paper advocates collaboration between universities and esports organizations and encourages flexibility and innovation.

## Data Availability

The raw data supporting the conclusions of this article will be made available by the authors, without undue reservation.

## Appendix A The semi-structured interviews

1. What opportunities exist in linking esport with academia?
2. What is the position of the Faculty of Physical Education and Sport/University? respectively in building new business models in the field of esport?
3. In what research areas can the Faculty of Physical Education and Sport/University contribute from its position as one of the actors within the esport ecosystem?
4. What specific groups of people involved in esport are lacking adequate education? What specific knowledge or skills do you have in mind?
5. How should particular groups be involved in the strategic management of the esport ecosystem?
6. Who do you think should lead the development and conception of esport?
7. What is the current level of esport infrastructure and institutional set-up in the country?
8. What is the level of influence and interest of unitary stakeholders within the esport ecosystem?
9. In your opinion, where is the biggest problem currently hindering the development of esport in the country?
10. What are the main barriers to the development of cooperation between the university environment and individual esport stakeholders?
